# Catheter-Associated Urinary Tract Infections in the Adult Patient Group: A Qualitative Systematic Review on the Adopted Preventative and Interventional Protocols From the Literature

**DOI:** 10.7759/cureus.16284

**Published:** 2021-07-09

**Authors:** Mohamed H Gad, Hesham H AbdelAziz

**Affiliations:** 1 Surgery, The Queen Elizabeth Hospital King's Lynn NHS Foundation Trust, King's Lynn, GBR; 2 Urology, Al Soliman Hospital, Port Said, EGY

**Keywords:** catheter-associated urinary tract infection, urinary tract infection, urinary catheterization, indwelling catheterization, asymptomatic bacteriuria, catheter-associated bacteriuria, intervention studies

## Abstract

Catheter-associated urinary tract infections (CA-UTIs) are among the most common nosocomial infections acquired by patients in health care settings. A significant risk factor for CA-UTIs is the duration of catheterization. To summarize the current strategies and interventions in reducing urinary tract infections associated with urinary catheters, use and the need for re-catheterization on the rate of CA-UTIs, we performed a systematic review. A rapid evidence analysis was carried out in the Medline (via Ovid) and the Cochrane Library for the periods of January 2005 till April 2021. The main inclusion criterion required to be included in this review was symptomatic CA-UTI in adults as a primary or secondary outcome in all the included studies. Only randomized trials and systematic reviews were included, reviewed, evaluated, and abstracted data from the 1145 articles that met the inclusion criteria. A total of 1145 articles were identified, of which 59 studies that met the inclusion criteria were selected. Studies of relevance to CA-UTIs were based on: duration of catheterization, indication for catheterization, catheter types, UTI prophylaxis, educational proposals and approaches, and mixed policies and interventions. The duration of catheterization is the contributing risk factor for CA-UTI incidence; longer-term catheterization should only be undertaken where needed indications. The indications for catheterization should be based on individual base to base cases. The evidence for systemic prophylaxis instead of when clinically indicated is still equivocal. However, antibiotic-impregnated catheters reduce the risk of symptomatic CA-UTIs and bacteriuria and are more cost-effective than other impregnated catheter types. Antibiotic resistance, potential side effects and increased healthcare costs are potential disadvantages of implementing antibiotic prophylaxis.

Multiple interventions and measures such as reducing the number of catheters in place, removing catheters at their earliest, clinically appropriate time, reducing the number of unnecessary catheters inserted, decrease antibiotic administration unless clinically needed, raising more awareness and provide training of nursing personnel on the latest guidelines, can effectively lower the incidence of CA-UTIs.

## Introduction and background

Catheter-associated urinary tract infection (CA-UTI) is defined as a urinary tract infection that occurs with the use of an indwelling urinary catheter. A prevalence survey from 2006 about hospital-acquired infections in acute hospitals in Ireland revealed that UTIs account for 22.5% in a hospital setting, of which 56.2% were catheter-related [1]. A urinary tract infection (UTI) is an infection to the epithelium of the urinary tract in response to the colonization of the pathogen. Urinary tract infections (UTIs) are one of the most common hospital community-acquired infections (HCAI), with up to 70-80% attributable to the presence of indwelling urinary catheters [2]. Between 10% to 25% of hospitalized patients, during their hospitalization, will receive indwelling urinary catheters, of whom 20% develop UTIs [3,4]. The risk of catheter-related infection increases by 5% each subsequent day the catheter remains in situ, with the risk increasing to 35% and 70% after seven and 14 days of indwelling catheterization, respectively. Around 50% of patients with indwelling catheters after 15 days of installation will develop UTIs, and almost 100% of the patients will develop UTI in one month [5]. Results of the 2009 pilot study for the European HCAI (HALT) study in long-term care facilities revealed that urinary tract infections accounted for 30% of the reported HCAIs and that almost half of all systemic antimicrobials were prescribed for an indication related to the urinary tract (48.9%) [6]. 

In line with the literature findings, it is clear that there is no standardization or even consensus among practitioners and hospitals/institutions regarding the protocols carried out of urinary catheter's insertion and maintenance. Regarding antibiotics prophylaxis, type of catheter to use, dwell time of the catheter, peri-urethral cleansing with anti-septic or sterile solutions etc., non-standardized practices in managing catheterized patients are noticed. A study by Conway et al. revealed that implementation protocol guidelines for CA-UTI prevention in the ICUs (intensive care units) is inadequate and insufficient, with 42% of ICUs reported having existing evidence-based practices (EBP) and policies for prophylaxis [7]. Therefore, there is a need for competent healthcare workers to set up and adhere to preventive and management protocols to reduce the probability of catheter-related infection. This article aims to provide a general overview of urinary catheterization and its association with UTI and preventative strategies by presenting the available results and recommendations in the literature.

## Review

Method 

A literature search was carried out in April 2021 in the Medline (via Ovid) and the Cochrane Library databases. Our searches used the following joint search term variations of the following Medical Subject Heading terms, specifically tailored for each database. The search terms- "urinary tract infections," "bacteriuria," "catheter," "indwelling catheter," "urinary catheterization," "asymptomatic bacteriuria," ''intervention studies"-were looked at both as free texts and MesH terms. We also evaluated the reference lists of articles, which provided us with further articles for consideration. Only full-text publications in English were considered. While catheter-associated asymptomatic bacteriuria was mentioned and compared to CA-UTI, it was decided not to be included. 

Study Selection 

**Figure 1:**
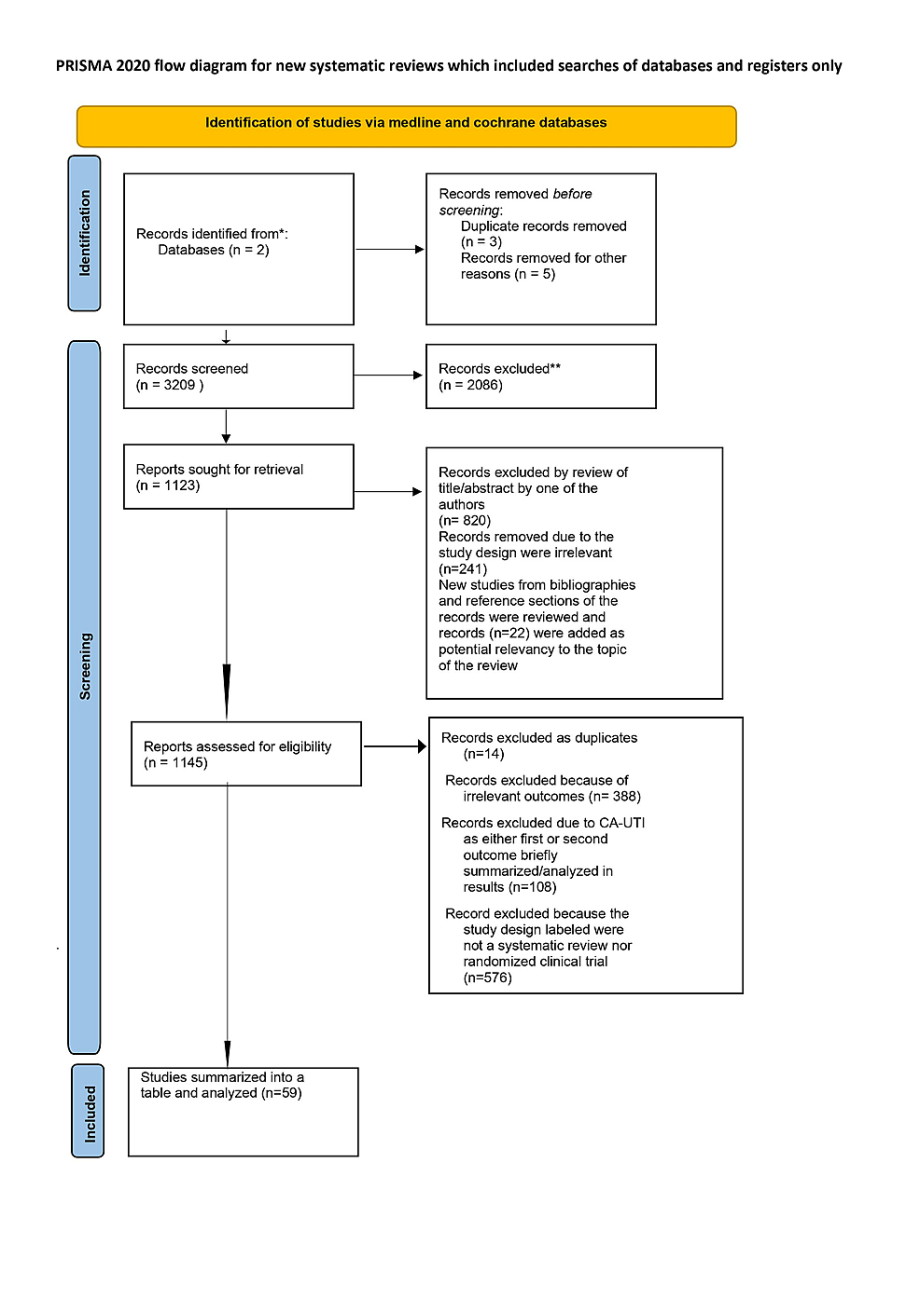
PRISMA 2020 flow diagram of the three phases of the systematic review. PRISMA 2020 flow diagram of the three phases of the systematic review [8]. For more information, visit: http://www.prisma-statement.org/prismastatement/flowdiagram.aspx

A rapid evidence analysis was carried out in the Medline (via Ovid) and the Cochrane Library for January 2005 to April 2021. The main inclusion criterion required to be included in this review was symptomatic catheter-associated UTI in adults as a primary or secondary outcome in all the included studies. Only randomized trials and systematic reviews were included in this systematic analysis. One thousand one hundred forty-five articles were identified, of which 1086 were excluded, as explained in figure 1. The final review is thus based on 59 articles. 

Data Extraction and Quality Assessment

The two authors of this review (HA and MG) independently reviewed and abstracted data from the 1145 articles that met the inclusion criteria. Extracted data included primary study objectives, patient population characteristics, inclusion criteria, terms and definitions used and quality issues. The data from the literature search were evaluated and shortlisted by the first author according to methodological/theoretical rigor and trustworthiness and data relevance on CA-UTI as a primary or secondary outcome. 

Study Characteristics for Inclusion

Our database search included publications published in the English language. We did not exclude studies based on the number of residents or patients included (gender, age, catheter type, use of antibiotic), duration of pre and post-intervention periods, study withdrawals, or whether follow-ups were done or not. 

Data Source and Searching the Literature 

The following data sources were searched: Ovid MEDLINE, Cochrane Library via Wiley and CINAHL. Only systematic search strategies were performed in the process of collecting the data (Figure 1). The first systematic search was conducted using the previously mentioned data sources to find searches associated with indications and need for catheterization, type of catheterization, duration of catheterization, infection prophylaxis, education programs, and interventions at reducing UTIs. The second systematic search was conducted in the described above data sources Ovid MEDLINE & Cochrane to identify RCT or studies to reduce UTI incidences use of antimicrobial coated catheters in settings such as hospitals, nursing homes, communities and rehabilitation units and spinal cord injury or orthopaedic programs, which do compromise a considerable population with chronic catheter needs. 

Study Selection & Data Extraction

A data collection instrument was adapted and used for characterization of the selected studies containing items such as the descriptors used, title, authors, area of work, year of publication, language, design, objectives, method, results, conclusion, recommendations, limitations and level of scientific evidence, among others. The three phases of this systematic review are detailed in figure 1 [8]. 

The first systematic search was filtered by title and abstract and applied a few exclusions (no symptomatic UTI/CA-UTI related outcomes were analyzed), identified duplicate studies, reviewed, and assigned potentially relevant studies and categorized them into groups such as review articles, clinical trials, comparative studies, and meta-analyses. Co-authors HA & MG filtered the records by title and abstract and reviewed the reference lists, of the included studies in this review, for additional relevant articles. Each author scored the studies, and the individually obtained results were later compared. Both authors' discrepancies in the scores were re-revised to ensure that the doubts concerning each studies inclusion were eliminated. Duplicates were removed. As exclusion criteria, the authors chose articles with non-relevancy to the issue of urinary catheterization, UTI or CA-UTI. 

The Jadad scale assessed the selected articles for evaluating the methodological quality of the selected RCT. The articles were graded from zero to five according to their methodological rigor and quality. One point is awarded for each of the following three questions: the description of randomization, the method of blinding, and withdrawals and dropouts. An extra point is attributed for each appropriately described randomization and blinding, up to a maximum of five points. A score of over three points constitutes an RCT of methodological rigor, and under three points were lower thoroughness. The extracted data from the 59 studies which made up this review are included in the results and discussion sections. 

*Outcomes* 

We researched studies including CA-UTI from the usage of an indwelling Foley urinary catheter or CA-UTI due to other catheter types such as intermittent or suprapubic catheters. 

Results

Analysis of the Literature Search 

The database search yielded 1145 results (Figure 1), of which 59 relevant studies were included in this review (Table 1). The included studies are grouped thematically: duration of catheterization (n = 9), type of catheterization (n = 13), assessing indication/necessity for catheterization (n = 2), maintenance and care of catheterized patients (n= 5), prophylactic measures (n = 17), preventative and/ or educational initiatives (n = 10), and studies with multiple interventions (n = 3). A total of 23 systematic reviews (including 6 Cochrane reviews) and 36 randomized, controlled trials (RCTs) were identified.

**Table 1:** Summary of Catheter-associated UTIs related reviews/randomized controlled trials Abbreviations: UTI: urinary tract infection.  CA-UTI: catheter-associated urinary tract infection. RCT: randomized controlled trial. S: systematic review. MA: metanalysis. n: number of patients. RR: risk ratio. OR: odds ratio. HR hazard ratio. p: p-value. CI: confidence interval.

First Author, Year, Citation	Study design	Studies’ objective(s)	Selection criteria of patients	Details of intervention	Main Results	Author’s exact conclusion	Notes
Studies on the route of administration and catheterization selection type							
Hakvoort et al., 2011 [9]	RCT/ multicenter	To compare clean intermittent catheterisation with transurethral indwelling catheterisation for the treatment of abnormal post‐void residual bladder volume (PVR) following vaginal prolapse surgery.	Patients older than 18 years experiencing abnormal PVR following vaginal prolapse surgery without or without the use of mesh.	Group A (45 patients): clean intermittent catherisation (CIC) for days 3 days. Group B (45 patients): transurethral indwelling catherization (TIC) for 3 days prophylactic antibiotics were given to all patients during surgery. A 14 French silicone transurethral indwelling catheter and a vaginal gauze were used after surgery. The catheter removal on the first postoperative day.	Group A: 5 (12%) Group B: 13 (33%) P = 0.03 in the CIC group (n = 45)	Clean catheterisation is preferable over indwelling catherisation for 3 days in the treatment of abnormal PVR following vaginal prolapse surgery	
Hälleberg Nyman et al., 2013 [10]	RCT/ cost analysis	to investigate differences between intermittent and indwelling urinary catheterisation in hip surgery patients in relation to nosocomial UTI and cost-effectiveness.	patients aged >50 undergoing hip fracture surgery or hip replacement surgery due to osteoarthritis	A (89 patients): intermittent urinary catheterisation B (93 patients): indwelling urinary catheterisation No antibiotic prophylaxis intraoperatively. Foley catheter 14 was used, and aseptic technique after preoperative antiseptic showering	UTI numbers: 8 in the intermittent catheterisation group, 8(9%) 10 in the indwelling catheterisation group, 10(11%) Absolute difference 2.4%, 95% CI -6.9-11.6%)	Both indwelling and intermittent methods could be appropriate in clinical practice. Both methods have advantages and disadvantages but by not using routine indwelling catheterisation, unnecessary catheterisations might be avoided in this patient group.	
Zhang et al., 2015 [11]	SR on RCTs & MA	To compare the rates of urinary tract infection (UTI) and postoperative urinary retention (POUR) in patients undergoing lower limb arthroplasty after either indwelling urinary catheterization or intermittent urinary catheterization.	patients undergoing total joint arthroplasty in the lower limb	Group A: indwelling catheterization Group B: intermittent catheterization	9 RCTs (on 1771 patients) showed no significant difference in the rate of UTIs between the two modes of catherization (P>0.05), RR 1.23 (0.85-1.76	Indwelling urinary catheterization with removal 24 to 48 hours postoperatively did not increase the risk of UTI.	
Hunter et al., 2013 [12]	SR	to examine research activity comparing suprapubic catheterization to any other method of chronic bladder emptying such as intermittent and indwelling catheterization in adults in relation to complications, patient satisfaction, and health-related quality of life (QoL).	Adult patients	Group A: Suprapubic catheters Group B: Urethral catheter and its types	the clinical evidence on UTI and long-term use of Suprapubic catheters showed no difference between urethral and SP catheters, no evidence that favors suprapubic.	Most studies focused on clinical urologic issues rather than patient understanding of suprapubic catheter management, satisfaction, stoma and skin care, or health related QoL.	
Dixon et al., 2010 [13]	RCT	To compare the use of intermittent urethral catherization with indwelling suprapubic catherization in women undergoing surgery for urodynamic stress incontinence or uterovaginal prolapse.	Women undergoing surgery for pelvic organ prolapse and/or stress urinary incontinence.	Group A (38 patients): suprapubic catheter, 48 hours postoperatively Group B (37 patients): intermittent urethral catheterization postoperative Three were withdrawn from study, leaving 36 women in each group	UTI incidence %: A: 9= 25% B: 13= 36% No significant difference in the rate of UTI between the two group	The use of intermittent catheterization following urogynaecological surgery is associated with a more rapid return to normal micturition and a shorter hospital stay, although the clinical significance of the difference is perhaps limited.	
Stekkinger et al., 2011 [14]	RCT	To compare the effect of suprapubic and transurethral catheterization on postvoid residual volumes (PRVs) after cystocele repair.	Women who underwent pelvic organ prolapse surgery including cystocele repair	Group A: suprapubic catheter (n = 64) Group: transurethral catheter (n = 62)	Urinary tract infections %: A: 9.3% B: 9.7% (P=0.93)	Suprapubic catherization was comparable to transurethral catherization in the prevention of postoperative voiding dysfunction after vaginal prolapse surgery, but it was associated with a higher rate of complications	
Healy et al., 2012 [15]	SR on RCT	comparing suprapubic catheterization and urethral catheterization in gynecologic populations.	women undergoing gynecologic surgery	12 RCTs included, N=1,300 patients Group A: Suprapubic Group B: Urethral	postoperative urinary tract infections: A: 20% B: 31% OR 0.31, 95% CI (0.185-0.512), P<.01 numbers support= the low association of suprapubic catherization to <.01.	Based on the best available evidence, no route for bladder drainage in gynecologic patients is clearly superior. The reduced rate of infective morbidity with suprapubic catheterization is offset by a higher rate of catheter-related complications and crucially does not translate into reduced hospital stay. As yet, there are insufficient data to determine which route is most appropriate for catheterization; therefore, cost and patient-specific factors should be paramount in the decision. Minimally invasive surgery may alter the requirement for prolonged postoperative catheterization.	
Kidd et al., 2015 [16]	Cochrane of RCT	To determine the advantages and disadvantages of alternative routes of short-term bladder catheterization.	short-term urethral catheterization uses in hospitalized adults	Group A: Suprapubic catheterization Group B: Indwelling catheterization 'short-term' was intended as duration of catheterization for 14 days (about 2 weeks) or less	RR 1.01, 95% CI 0.61 to 1.69; 5 trials, 575 participants	Suprapubic catheters reduced the number of participants with asymptomatic bacteriuria, recatheterisation and pain compared with indwelling urethral. The evidence for symptomatic urinary tract infection was inconclusive. For indwelling versus intermittent urethral catheterisation, the evidence was inconclusive for symptomatic urinary tract infection and asymptomatic bacteriuria. No trials reported pain.The evidence was inconclusive for suprapubic versus intermittent urethral catheterisation. Trials should use a standardised definition for symptomatic urinary tract infection. Further adequately-powered trials comparing all catheters are req to investigate differences between intermittent and indwelling urinary catheterisation in hip surgery patients in relation to nosocomial UTI and cost-effectiveness. uired, particularly suprapubic and intermittent urethral catheterisation.	This is an update of Niël-Weise’s 2005 Urinary catheter policies for short-term bladder drainage in adult review.
Van den Eijkel et al., 2006 [17]	SR on RCTs	To review the effect of catheter valves compared to free drainage into a bag for patients with indwelling urinary catheters.	Patients older than 16 years old.	Group A: catheter valve Group B: catheter bag drainage system	Only one study out of the two RCTs assessed for UTI	No statistically differences for UTIs in the comparison of catheter valve vs standard continuous drainage bag were demonstrated	the one study was conducted by (Wilson C, Sandhu SS, Kaisary AV. A prospective randomized study comparing a catheter-valve with a standard drainage system. Br J Urol. 1997)
Darouiche et al., 2006 [18]	RCT	To assess the impact of using the StatLock securing device on symptomatic catheter-related urinary tract infection (UTI).	Adult patients with spinal cord injury diagnosed with neurogenic bladder and needed a long-term indwelling transurethral or suprapubic bladder catheter	Experimental Group A (60 patients): indwelling bladder catheters secured in place by using the StatLock device. Control Group B (58 patients): traditional methods that included tape, Velcro strap, Cath-Secure or none.	Symptomatic UTI number (%): A: 8/60= (13.3%) B: 14/58= (24.1%) (P = 0.16; RR = 0.55, 95% confidence interval: 0.25-1.22).	45% reduction in the rate of symptomatic UTI in patients who received the StatLock securing device is clinically relevant and prompts further investigations.	
Gong et al., 2017 [19]	RCT	To determine the effect of clamping the indwelling urinary catheter before its removal on bladder reconditioning.	patients with cervical cancer after type C radical hysterectomy.	Group A (70 patients): intermediately clamp indwelling urinary catheters for 48 hours before removing it Group B (128 patients): indwelling urinary catheters removal without clamping	incidence of urinary tract infection Group A: 22·9% Group B: 20·3% showed no significant differences between the two groups	Bladder reconditions through indwelling urinary catheter clamping may not restore bladder function in patients after radical hysterectomy.	
Fernandez et al., 2005 [20]	SR on RCT	To determine the effects of clamping short-term indwelling urethral catheters before removal on the incidence of urinary tract infection, time to first void, voiding dysfunction, incidence of recatheterization, and the length of hospital stay.	clamping before removal of short-term indwelling urethral catheters in people in of all ages	1 trial out of the three trials discussed about CA-UTI: Group A: indwelling catheter removed within 24 hours after free drainage Group B: indwelling catheter removed within 72 hours (about 3 days) after free drainage Group C: indwelling catheter removed within 24 hours + bladder training	There were 106 women included in the one trial on CA-UTI, UTI incidence % were: Group A: 3/37= (8%) Group B: 6/36= (16.6%) Group C: 3/33= (9%)	The evidence for clamping indwelling urethral catheters before removal remains equivocal. Given the current state of evidence, procedures relating to clamping of indwelling urinary catheters should not be initiated. Until stronger evidence becomes available, however, practices relating to clamping indwelling urethral catheters will continue to be dictated by local preferences and cost factors.	
Wang et al., 2016 [21]	SR on RCT	To examine the necessity of clamping before removal of an indwelling urinary catheter in short-term patients.	surgical inpatients with indwelling urinary catheter up to 14 days	Group A: Regular clamp on urinary catheter clamped off before removal Group B: leave the urinary catheter on free draining until removal	In four studies reviewed, Catheter clamping prior to removal was not necessary for the short-term patient. No significant difference between clamping and unclamping were found in recatheterization risk, nor rate of urinary tract infection. OR 0.76, 95% CI (0.33, 1.73)	This review indicated that bladder training by clamping prior to removal of urinary catheters is not necessary in short-term catheter patients. In addition, clamping carries the risk of complications such as prolonging urinary catheter retention and urinary tract injury. Further investigation requires higher quality methodologies and more diverse study designs.	
Duration of catheterization and dwell time							
Alessandri et al., 2006 [22]	RCT	Assessing immediate versus delayed catheter removal.	randomly assigned who underwent hysterectomy for various benign diseases.	three groups (32 women for each group). Group A: immediate removal of the catheter in the operating room. 2. Group B: removal of catheter at 6 h after the operation. 3. Group C: removal of catheter at 12 h after the operation. All patients received a single dose of antibiotic prophylaxis before hysterectomy. 16F latex catheters with a 10 ml balloon were used as well.	Symptomatic urinary infection in the three groups (%): A: 1/32 = 3.1 B: 4/30 = 13.3 C: 5/32 = 15.6	There could be an association between necessity of re-catheterization and the type of surgery (VH) or the type of anesthesia (spinal). Despite re-catheterization rate, early removal of indwelling catheters immediately after uncomplicated hysterectomy seems to decrease first ambulation time and hospital stay.	
Sekhavat et al., 2008 [23]	RCT	To assess whether immediate removal of an indwelling catheter after anterior colporrhaphy influences the rate of re-catheterisation and symptomatic urinary tract infections.	90 women aged between 40 and 50 years who underwent anterior colporrhaphy.	The women were divided into two groups: A: removal immediately after surgery B: removal at least 24 h after OP n=45 for both groups	UTI percentage (supported with positive urine culture): Group A: 4,5% Group B: 15,5% P= 0,01	Early removal of an indwelling catheter immediately after anterior colporrhaphy was not associated with adverse events and an increased rate of re-catheterization. In this group, symptomatic urinary tract infection was significantly lower. Moreover, early removal of indwelling catheters immediately after operation seemed to decrease the ambulation time and hospital stay.	
Chai et al., 2011 [24]	RCT	To assess whether early or immediate removal of a 12F in-dwelling Foley catheter after total abdominal hysterectomy affects the level of subjective pain assessment postoperatively.	Women undergoing total abdominal hysterectomy for various benign gynecological diseases after counseling about available alternative treatments.	Two designated groups: Group A (35 patients): catheter removed immediately post-surgery Group B (35 patients): catheter removed on a postoperative day one, i.e. 24 hours after the operation Latex 12F with a 10ml balloon Foley catheter under aseptic technique and catheter urine were collected for microscopy and culture. Routine prophylactic antibiotics were not given.	symptomatic urinary tract infection, n (%): A: 1 = (2.9) B: 3 = (8.6)	There are pros and cons regarding the policy of one-day in-dwelling catheterization compared to immediate catheter removal.	
Ahmed et al., 2014 [25]	RCT	assess whether immediate (0h), intermediate (after 6h) or delayed (after 24h) removal of an indwelling urinary catheter after uncomplicated abdominal hysterectomy can affect the rate of re-catheterization due to urinary retention, rate of urinary tract infection, ambulation time and length of hospital stay.	221 women underwent total abdominal hysterectomy for benign gynaecological diseases and were randomly distributed into three groups. On the morning of surgery, all patients received a single dose of prophylactic antibiotic (ceftriaxone 1 g) intramuscularly.	Group A (73 patients): catheter removed immediately after surgery (0 h) Group B (81 patients): catheter removed 6 h post-surgery Group C (67 patients): catheter removed 24 h post-surgery size 12 latex Foley's catheter were used.	Symptomatic UTI, n (%): Group A: 1 = (1,4) Group B: 3 = (3,7) Group C: 10 = (14,9) (p=0.008)	Removal of the urinary catheter 6h postoperatively appears to be more advantageous than early or late removal in cases of uncomplicated total abdominal hysterectomy.	
El-Mazny et al., 2014 [26]	RCT	To compare immediate and 12h postoperative removal of urinary catheter after elective cesarean section.	300 eligible women admitted for primary or repeat elective cesarean section.	The women were randomized into two equal groups: Group A (n = 150): catheter was removed immediately after the procedure Group B (n=150): the catheter was removed 12h postoperatively. Foley urethral catheter was used and antibiotic prophylaxis was given to all patients.	Postoperative urinary complications %: 1-Dysuria: Group A: 11 (7.3) Group B: 24 (16.0) p value: 0.030 2-Burning on micturition: Group A: 4 (2.7) Group B: 15 (10.0) p value: 0.016 3-Urinary frequency: Group A: 3 (2.0) Group B: 12 (8.0) p value: 0.031	Immediate removal of urinary catheters after elective cesarean section is associated with a lower risk of urinary infection and earlier postoperative ambulation.	
Bray et al., 2017 [27]	RCT	To determine if indwelling catheterisation is necessary after vaginal surgery for pelvic organ prolapse.	immediate post-operative removal of catheter compared to a suprapubic catheter (SPC) after vaginal prolapse surgery via the vaginal route.	Two groups were created: Group A (29 patients): Suprapubic catheter until 48 h (day 2) postoperative. Group B (31 patients): immediate removal of one dose of intraoperative prophylactic antibiotics administered.	Rate of symptomatic bacteriuria (n): A: 15 B: 5 (p<0.01)	Early removal of a catheter reduces urinary tract infection and significantly decreases hospital stay. Such a policy should result in improved patient satisfaction and reduced hospital costs.	
Weemhoff et al., 2011 [28]	RCT	compare the number of temporary catheter replacements and urinary tract infections after indwelling catheterization for 2 versus 5 days following an anterior colporrhaphy.	246 patients with cystocele undergoing an anterior colporrhaphy were eligible	Two groups were assigned Group A: catheter for 2 days Group B: catheter for 5 days	Urinary tract infection percentage: Group A: 22%, proven by a culture with >105 colony forming units per milliliter, Group B: 37% OR= 0.5 (CI 0.3-0.9, p = 0.02)	Removal of an indwelling catheter after 2 versus 5 days following anterior colporrhaphy is associated with more temporary catheter replacements, but fewer urinary tract infections and a shorter hospital stay.	
Liang et al., 2009 [29]	RCT	to assess the impact of bladder catheterization on the incidence of postoperative urinary tract infection (UTI) and urinary retention (PUR) following laparoscopic-assisted vaginal hysterectomy (LAVH)	patients with benign gynecologic disease scheduled for LAVH	Group A (50 patients): no catheter Group B (50 patients): indwelling bladder catheter for 1-day Group C (50 patients): indwelling bladder-catheter for 2 days	UTI: Group A: 2 (4%) Group B: 3 (6%) Group C: 9 (18%) (p= 0.034)	although the incidence of catheter-associated UTI following LAVH was confounded by the use of prophylactic antibiotics in our study, the data suggest that the duration of catheterization was the most important predictor for postoperative adverse urinary events. Short term indwelling catheterization increased the incidence of UTI but decreased the incidence of PUR among patients undergoing LAVH.	
Fernandez et al., 2006 [30]	A systemic review on RCT and NRCT	assess the effect of duration of catheterization on urinary retention.	8 trials were conducted on patients of different ages and in surgical relevance.	Different durations of catheterization were set before the removal of short-term indwelling urethral catheters.	Immediate versus within 24-48 hours versus after 48-hour removal of indwelling catheters Four of the trials out of the eight proved no significant differences statistically in patient outcome with an incidence of UTIs after TURP (RR 0.55, 95% CI 0.30 to 1.03). 1 trial of patients who had their indwelling urethral catheters removed 5 days after rectal resection reported a higher incidence of urinary tract infection, in comparison to the patients who had their indwelling urethral catheters removed 1 day after surgery (RR 0.48, 95% CI 0.27 to 0.85).	No significant differences in patient outcome were found, but the timing of catheter removal is a balance between avoiding infection by early removal and circumventing voiding dysfunction by later removal. Shorter catheterizations appear to reduce the mean length of hospital stay.	
Maintenance and care of catheterized patients							
Ercole et al., 2013 [31]	SR	to seek the best evidence available in the literature concerning the knowledge produced and related to the techniques of intermittent and indwelling urinary catheterization	28 RCT & 9 SR	Clean or sterile technique, sterile water vs Intermittent catheter vs indwelling catheter	the infection rate in the urinary tract does not vary whether using sterile or non-sterile methods. The use of an intermittent catheter with a clean technique results in low rates of complications or infections compared to the use of an indwelling catheter. The removal of the catheter up to 24 hours after surgery and the use of an antimicrobial-impregnated are favoured.	there are controversies in relation to the periurethral cleansing technique, the type of material the catheter is made of, and some procedures for the maintenance and removal of the catheter.	
Cao et al., 2018 [32]	SR and Network MA of RCT	To evaluate the best cleaning methods of urethral cleaning versus disinfection for the prevention of CA-UTIs through conducting a network meta-analysis of the literature using the Bayesian method.	33 studies (6490 patients) patients >18 years with indwelling urinary catheters (IDC)	Group A: cleaned the meatal, peri-urethral, or perineal areas before IDC insertion or intermittent catheterization, or during routine meatal care using an antiseptic such as iodine, chlorhexidine, nitrofurazone, etc. Group B: cleaned with non-medicated agents such as sterile water, tap water, saline, etc.	7 different methods of urethral cleaning versus disinfection were eligible for inclusion, no heterogeneity in the incidence of CA-UTIs documented in the studies The 7 urethral cleaning methods versus disinfection resulted in (P > 0.05) for both, no difference in the incidence of CA-UTIs. Chlorhexidine ranked first in the results of the Bayesian analysis and is recommended for preventing CA-UTIs.	Current evidence suggests that there are no significant differences among different urethral cleaning versus disinfection methods with regard to CA-UTI incidence rates.	
Cheung et al., 2008 [33]	RCT	to compare the risk of acquiring symptomatic urinary tract infections through the conventional practice of using 0.05% chlorhexidine gluconate versus sterile water for periurethral cleansing before insertion of an indwelling urinary catheter.	Adults with a long-term indwelling urinary catheters in a nursing setting	A: (12 patients)- 0.05% chlorhexidine gluconate B: (8 patients)- sterile water Latex catheters were used in this study. Urine specimens for culture were collected 4 times for each subject within 2 weeks.	none of the subjects in the 2 groups developed symptomatic bacteriuria.	Using sterile water to clean the periurethral area before catheterization among home care patients will not increase the risk for urinary tract infections.	
Sinclair et al., 2011 [34]	SR	To determine if certain washout regimens (including no washout) are better than others in the management of long-term indwelling urinary catheters in adults.	adults (16 years and above) in any setting (hospital, nursing/residential home, community) with an indwelling urethral or suprapubic catheter in place for more than 28 days.	Compared various washout regimens (i.e., washout vs. no washout, saline or acidic solutions, saline versus acidic solution versus antibiotic solution)	No difference between all indicated washout regimens in terms of symptomatic urinary tract infections were reported	The data from five trials comparing differing washout policies were sparse and trials were generally of poor quality or poorly reported. The evidence was too scant to conclude whether or not washouts were beneficial. Further rigorous, high-quality trials with adequate power to detect any benefit from washout rather than no washout being performed are required in the first instance. After that, trials comparing different washout solutions, washout volumes, frequencies/timings, and routes of administration are needed.	
Shepherd et al., 2017 [35]	SR on Cochrane and quasi-RCTs	To determine if certain washout regimens are better than others in terms of effectiveness, acceptability, complications, quality of life and critically appraise and summarise economic evidence for the management of long-term indwelling urinary catheterization in adults.	Adults older than 16 years with the condition of having an indwelling urethral or suprapubic catheter for more than 28 days	Various catheter washout policies were reviewed (e.g. washout versus no washout, washout solution versus another, type of catheter washout solution versus another, frequency, route of administration) A. Any washout versus no washout. B. saline washout versus no washout. C. citric acid washout versus no washout:	Symptomatic UTI patient number was 0.0 [0.0, 0.0] for any washout versus no washout saline washout versus no washout citric acid washout versus no washout	Data from seven trials that compared different washout policies were limited, and generally, of poor methodological quality or were poorly reported. The evidence was not adequate to conclude if washouts were beneficial or harmful. Further rigorous, high-quality trials that are adequately powered to detect benefits from washout being performed as opposed to no washout are needed. Trials comparing different washout solutions, washout volumes, and frequencies or timings are also needed.	This is an update of a review (Hagen 2010, Washout policies in long-term indwelling urinary catheterization in adults.) published in 2010. Hence, the 2010 review by Hagen et al was excluded
Assessing indications/necessity for catheterization							
Nasr et al., 2009 [36]	RCT, multicenter	To prospectively investigate the effects on urinary tract infection (UTI) of indwelling urinary catheter placement during cesarean delivery.	Patients during caesarian delivery	Group A (n=210): non catheterized Group B (n=210): Catheterized, control	UTI was greater in the catheterization group (P<0.001)	Non-placement of an indwelling urinary catheter during cesarean was more convenient to women with no increase in intraoperative complications or urinary retention. Indwelling catheter placement in hemodynamically stable patients proved not to be beneficial in this study.	
Li et al., 2011 [37]	SR	To assess whether the necessity to place indwelling urinary catheters routinely in caesarean section, and examine UTIs, urinary retention, intraoperative difficulties, operative complications, as well as others	Two RCTs and an NRCT were reviewed	Group A: indwelling catheterization pre-surgery. Group B: No catheterisation pre-surgery	The non catheirzed group had a lower incidenceof UTIs in both the two RCT and one NRCT : [RR 0.08; 95% CI 0.01, 0.64] and [RR 0.10; 95% CI 0.02, 0.57 ] respectively	The non-use of indwelling urinary catheters in a caesarean section is associated with fewer UTIs and no increase in either urinary retention or intra-operative difficulties. Our results suggest that the routine use of indwelling urinary catheters for caesarean delivery in haemodynamically stable patients is not necessary, and can be harmful. However, better and larger randomised trials are needed to confirm these findings.	
Studies on UTI prophylaxis							
Lam et al., 2014 [38]	SR Cochrane on RCT	To compare the effectiveness of different types of indwelling urethral catheters in reducing the risk of UTI and to assess their impact on other outcomes in adults who require short-term urethral catheterisation in hospitals.	hospitalized patients	A: Antiseptic-coated indwelling urethral catheters versus standard indwelling urethral catheters B: Antimicrobial-impregnated indwelling urethral catheters versus standard indwelling urethral catheters C: Antimicrobial-impregnated indwelling urethral catheters versus antiseptic-coated indwelling urethral catheters D: One type of standard indwelling urethral catheter versus another type of standard indwelling urethral catheter	CA-UTI: A: RR 0.99, 95% CI 0.85 to 1.16 B: RR0.84, 95%CI 0.71 to 0.99 C: RR 0.84, 95% CI 0.71 to 1.00 D: Trials included the standard catheter did not measure symptomatic CA-UTI.	Silver alloy-coated catheters were not associated with a statistically significant reduction in symptomatic CA-UTI, and are considerably more expensive. Nitrofurazone-impregnated catheters reduced the risk of symptomatic CA-UTI and bacteriuria, although the magnitude of reduction was low and hence may not be clinically important. However, they are more expensive than standard catheters. They are also more likely to cause discomfort than standard catheters.	
Lusardi et al., 2013 [39]	SR Cochrane on RCT	To determine if certain antibiotic prophylaxes are better than others in terms of prevention of urinary tract infections, complications, quality of life and cost-effectiveness in short-term catheterisation in adults.	789 adult patients requiring short-term urethral and supra-pubic catheterisation (up to and including 14 days) in the hospital were included.	Proposed interventions were: 1. antibiotic prophylaxis versus no prophylaxis 2. antibiotic prophylaxis with antibiotic A versus giving antibiotic prophylaxis with antibiotic B 3.antibiotic prophylaxis at catheterisation only versus antibiotic prophylaxis throughout the catheterisation period. In addition, the route of administration (oral or intravenous, but not topical) was considered.	UTI as a result from antibiotic prophylaxis versus no prophylaxis (based on 1 trial and 90 patients): RR 0.20 [0.06, 0.66]	The limited evidence indicated that receiving prophylactic antibiotics reduced the rate of bacteriuria and other signs of infection, such as pyuria, febrile morbidity and gram-negative isolates in patients' urine, in surgical patients who undergo bladder drainage for at least 24 hours postoperatively. There was also limited evidence that prophylactic antibiotics reduced bacteriuria in non-surgical patients.	
Pickard et al., 2012 [40]	RCT, multicenter	Do antimicrobial catheters reduce the rate of symptomatic urinary tract infection (UTI) during short-term hospital use and is their use cost-effective for the UK NHS?	Patients (≥ 16 years of age) requiring temporary urethral catheterisation for a maximum of 14 days as part of their care from elective surgery.	Group A (n = 2153): nitrofurazone-impregnated silicone Group B (n = 2097): silver alloy-coated latex hydrogel Group C (n = 2144): control group, polytetrafluoroethylene (PTFE) coated catheter	Randomized symptomatic antibiotic-treated UTI within 6 weeks: Nitrofurazone vs control PTFE: OR 0.81 (0.65 to 1.01); p=0.031 Silver alloy vs Control PTFcE: OR 0.96 (0.78 to 1.19); p=0.69	The trial estimate of clinical effectiveness for nitrofurazone-impregnated catheters was less than the pre-specified minimum absolute risk difference that we considered important (-3.3%), and the surrounding CI included zero, indicating that any reduction in catheter-associated UTI was uncertain. Economic analysis, although associated with uncertainty, suggested that nitrofurazone-impregnated catheters may be cost-effective for the NHS. The trial ruled out the possibility that silver alloy-coated catheters might reach the pre-set degree of clinical effectiveness and that their use was unlikely to be cost-effective. These findings should be considered by patients, clinicians and healthcare policy-makers to determine whether or not a change in practice is worthwhile. Future research should be aimed at determining the minimum clinically important difference in terms of CA-UTI prevention in comparative trials, and to identify reliable methods which can detect the impact of the intervention on quality of life and other drivers of cost, when the intervention is a subsidiary part of overall treatment plans.	
Bonfill et al., 2017 [41]	RCT, 14 hospitals ( ESCALE trial)	to assess the efficacy of antiseptic silver alloy-coated urinary catheters for preventing catheter-associated urinary tract infections.	Men or women with traumatic or medical SCI, aged ≥18 years, requiring an indwelling urinary catheter for at least 7 days.	Group A (n=243): Antiseptic silver alloy-coated silicone urinary catheters. Group B (n=246): silicone or silicone-latex catheters.	symptomatic UTI ratio: Group A: 18 (7.41%) Group B: 19 (7.72%) (odds ratio [OR] 0.96 [0.49-1.87]). The adjusted analysis revealed no change in the results.	The results of this study do not support the routine use of indwelling antiseptic SAC silicone urinary catheters in patients with SCI. However, UTIs associated to long-term urinary catheter use remain a challenge and further investigations are still needed.	
Jahn et al., 2012 [42]	RCT	To compare the incidence of catheter-associated bacteriuria with a noble metal alloy-coated latex catheter or a non-coated silicone catheter in patients undergoing elective orthopaedic surgery with short-term catheterization and to identify risk factors for bacteriuria and catheter-associated urinary tract symptoms.	Patients undergoing elective orthopaedic surgery	Group A (n=222): Noble metal alloy-coated latex. Group B (n=217): silicone Foley catheter. Catheter size was 12 Ch for both catheter groups.	Number of patients with catheter-associated urinary tract symptoms Group A: 22.1 Group B: 22.9 P= 0.849	This study confirmed previous results that the noble metal alloy coating significantly reduces the risk of catheter-associated bacteriuria in short-term catheterization (1-3 days). Female gender and obesity were significant risk factors for developing bacteriuria, while the use of an open drainage system and insertion of the catheter on the ward were not.	
Beattie et al., 2011 [43]	SR of SR and RCT	To determine whether there was enough evidence to conclude that silver alloy urinary catheters reduce catheter-associated urinary tract infections compared with silicone or latex urinary catheters in adult inpatients.	short-term hospitalized adult patients with < 2 weeks catheter use	11 total studies: 6 SR/MA & 5 RCT. silver-alloy urinary catheters versus silicone or latex urinary catheters in adult inpatients.	No study was able to definitively conclude that silver-alloy urinary catheters reduce CA-UTI in short term hospitalized patients.	The collective evidence divulged an emerging pattern favouring the efficacy of silver-alloy urinary catheters to reduce catheter-associated urinary tract infections. Owing to the poor quality of some individual studies included in other systematic reviews and the inability to carry out meta-analysis because of significant heterogeneity, definitive conclusions cannot be drawn from the study.	The inclusion of this review was contemplated due to the authors’ inability to carry out meta-analysis due to the heterogenicity of the data.
Johnson et al., 2008 [44]	RCT	To assess currently marketed antimicrobial urinary catheters for preventing catheter-associated urinary tract infection (UTI).	Total of 13392 patients in 12 trials were selected. nitrofurazone-coated or silver alloy-coated antimicrobial urinary catheter use for less than 30 days	catheters that were included: nitrofurazone coated silicone, silver hydrogel silicone, silver hydrogel–coated latex, silver hydrogel–co,	All trials suggested protection against bacteriuria with test catheter use. nitrofurazone-coated silicone (n = 3) or silver-coated latex (n = 9) catheters with silicone or latex catheters. No study addressed symptomatic UTI.	According to fair-quality evidence, antimicrobial urinary catheters can prevent bacteriuria in hospitalized patients during short-term catheterization, depending on antimicrobial coating and several other variables. Older data probably lack current relevance. Cost implications and effects on infectious complications remain undefined.	
Pfefferkorn et al., 2009 [45]	RCT	To assess whether antibiotic prophylaxis at urinary catheter removal reduces the rate of urinary tract infections	239 patients undergoing elective abdominal surgery.	Group A (n =103): antibiotic prophylaxis, 3 doses of trimethoprim-sulfamethoxazole at urinary catheter removal Group B (n= 102): without antibiotic prophylaxis	Symptomatic UTI: Group A: 5/103= 4.9% Group B: 22/102= 21.6% (p= < 0.001) absolute risk reduction 16.7%. the relative risk reduction 77.5%.	Antibiotic prophylaxis with trimethoprim-sulfamethoxazole on urinary catheter removal significantly reduces the rate of symptomatic urinary tract infections and bacteriuria in patients undergoing abdominal surgery with perioperative transurethral urinary catheters.	
Dieter et al., 2014 [46]	RCT	To evaluate whether nitrofurantoin prophylaxis prevents postoperative urinary tract infection (UTI) in patients receiving transurethral catheterization after pelvic reconstructive surgery.	participants undergoing pelvic reconstructive surgery were randomized	Two groups were randomized during catheterization: Group A (81 patients): 100 mg nitrofurantoin once daily during catheterization Group B (78 patients): placebo was given once daily during catheterization.	22% UTI with nitrofurantoin 13% UTI with placebo RR 1.73, 95% CI 0.85-3.52, P=.12	Prophylaxis with daily nitrofurantoin during catheterization does not reduce the risk of postoperative UTI in patients receiving short-term transurethral catheterization after pelvic reconstructive surgery.	
Marschall et al., 2013 [47]	SR and MA RCT and non-RCT	To clarify whether antibiotic prophylaxis at the time of urinary catheter removal confers a benefit in terms of preventing subsequent symptomatic urinary tract infections.	All adults requiring short-term urinary urethral and supra-pubic catheterisation (up to and including 14 days) in hospital	A: antibiotic prophylaxis B: no antibiotic prophylaxis	Antibiotic prophylaxis was associated with benefit to the patient, with an absolute reduction in risk of urinary tract infection of 5.8% between intervention and control groups. The risk ratio was 0.45 (95% confidence interval 0.28 to 0.72). The number needed to treat to prevent one urinary tract infection was 17 (Confidence interval 12 to 30).	Patients admitted to hospitals who undergo short term urinary catheterization might benefit from antimicrobial prophylaxis when the catheter is removed as they experience fewer subsequent urinary tract infections. Potential disadvantages of more widespread antimicrobial prophylaxis (side effects and cost of antibiotics, development of antimicrobial resistance) might be mitigated by the identification of which patients are most likely to benefit from this approach.	
Van Hees et al., 2011 [48]	RCT	investigated the effects of a single dose antibiotic regimen, before removing urinary catheters, on the occurrence of significant bacteriuria (SBU) and UTI.	Patients scheduled to undergo major surgery, such as an abdominal operation or hip surgery	Group A: co-trimoxazole (960 mg) (n= 46) Group B: ciprofloxacin (500 mg)) (n = 43) Group C: placebo (n = 51) The three groups were administered 2 hours before catheter removal	Symptomatic UTI incidence (%): A: 1/31 (3%) B: 0/24 (0%) C: 1/36 (3%)	our results do not support antibiotic prophylaxis for urinary catheter removal in non-genitourinary surgical patients.	
Berrondo et al., 2018 [49]	RCT	To evaluate the role of antibiotic prophylaxis with oral ciprofloxacin prior to urinary catheter removal after radical prostatectomy in preventing urinary tract infection (UTI).	Patients undergoing radical prostatectomy. One hundred seventy-five patients were enrolled	Group A: antibiotic prophylaxis group (2 doses of oral ciprofloxacin prior to urinary catheter removal) Group B: control group (no antibiotics given prior to urinary catheter removal)	Eighteen patients (7.41%) in the experimental group and 19 in the control (7.72%) group had a symptomatic UTI (odds ratio [OR] 0.96 [0.49-1.87]).	In this prospective, randomized, controlled trial, the use of antibiotic prophylaxis with oral ciprofloxacin prior to urinary catheter removal after radical prostatectomy did not decrease the rate of UTI and was not associated with an increased incidence of C diff enterocolitis.	
Foxman et al., 2015 [50]	RCT	To test the therapeutic efficacy of cranberry juice capsules in preventing UTI post-surgery.	Women with elective gynecologic surgery	Group A: cranberry capsules two times a day, for 6 weeks postoperatively Group B: placebo capsules All study participants received a prophylactic intravenous antibiotic administration preoperatively, including urinary catheter insertion (as per hospital protocol).	The occurrence of UTI %: A: 15/80 =19% B: 30/80 =38%; OR=0.42; 95% CI: 0.18, 0.94 p=0.008	Among women undergoing elective benign gynecologic surgery involving urinary catheterization, the use of cranberry extract tablets during the postoperative period reduced the rate of UTI by half.	
Gunnarsson et al., 2017 [51]	RCT	to investigate whether intake of cranberry juice concentrates pre-operatively decreases the incidence of postoperative UTIs in hip fracture patients that received a urinary catheter.	227 Female patients 60 years and older, with hip fractures.	Group A (n=50): two capsules of 550 mg of cranberry powder. Group B (n=61): placebo capsules. Both groups receive the capsules daily, from admission, until 5 days postoperatively. Urine cultures were obtained at admission, 5 and 14 days postoperatively. In addition, Euro Qual five Dimensions assessments were performed and patients were screened for UTI symptoms.	Number of patients with positive culture at either day 5 or day 14 postoperatively: A:19/50= (38%) B:23/61= (38%) (p=0.975, RR 0.988, 95% CI 0.457– 2.135)	Cranberry concentrate does not seem to effectively prevent UTIs in female patients with hip fracture and indwelling urinary catheters.	
Niel-Weise et al., 2005 [52]	SR Cochrane and quasi-RCTs	To determine if certain antibiotic policies are better than others in terms of prevention of urinary tract infections, complications, quality of life and cost‐effectiveness in short‐term catheterised adults.	All adults requiring short‐term urethral catheterization (up to and including 14 days) in hospital for urine monitoring, investigations, acute retention problems, acute incontinence problems and after surgery. These include those suffering from general medical problems, acute illness, urinary retention and following surgery.	The interventions considered were: antibiotic prophylaxis (continuous use), use of antibiotics if clinically indicated (e.g. pain, fever) and use of antibiotics if microbiologically indicated (growth of bacteria from a specimen of urine in the absence of clinical symptoms, the density of bacteria taken as positive as defined by the trialists).	Only one trial focused on symptomatic urinary tract infection. It showed a significantly lower rate in the group receiving prophylactic antibiotics, but the observation was based on only 16 cases of infection (RR 0.20, 95% CI 0.06 to 0.66, Comparison 01.01).	There was weak evidence that antibiotic prophylaxis compared to giving antibiotics when clinically indicated reduced the rate of symptomatic urinary tract infection in female patients with abdominal surgery and a urethral catheter for 24 hours. The limited evidence indicated that receiving antibiotics during the first three postoperative days or from postoperative day two until catheter removal reduced the rate of bacteriuria and other signs of infection such as pyuria and gram‐negative isolates in patients urine in surgical patients with bladder drainage for at least 24 hours postoperatively. There was also limited evidence that prophylactic antibiotics reduced bacteriuria in non‐surgical patients.	
Cardenas et al., 2011 [53]	RCT	To investigate whether intermittent catheterization with a hydrophilic coated catheter delays the onset of the first symptomatic urinary tract infection (UTI) and reduces the number of symptomatic UTIs in patients with acute spinal cord injury (SCI) compared with IC with standard, uncoated catheters.	224 subjects with traumatic SCI of less than 3 months duration who use intermittent catheterization. The duration of the study included 2 periods: 1.institutional period (in acute care or a rehabilitation unit) 2.community period (after discharge from the hospital or rehabilitation unit).	Group A (108 patients): hydrophilic-coated, polyurethane Nelaton (SpeediCath) catheter was a sterile, ready-to-use. The coating consists mainly of polyvinyl-pyrrolidone. Group B (116 patients): polyvinyl chloride uncoated (Conveen) catheters.	Total UTI/total months ratio: Group A: 99/206.7 = 0.479 Group B: 167/349.2 = 0.478	The use of a hydrophilic-coated catheter for IC is associated with a delay in the onset of the first antibiotic-treated symptomatic UTI and with a reduction in the incidence of symptomatic UTI in patients with acute SCI during the acute inpatient rehabilitation. Using a hydrophilic-coated catheter could minimize UTI-related complications, treatment costs, and rehabilitation delays in this group of patients, and reduce the emergence of antibiotic-resistant organisms.	
Fasugba et al., 2017 [54]	SR on RCT	To undertake a systematic review of the literature and meta-analysis of studies investigating the effectiveness of antiseptic cleaning before urinary catheter insertion and during catheter use for prevention of CA-UTIs.	14 studies on patients requiring short or long-term indwelling urethral catheter or intermittent catheterisation.	Group A: antiseptic catheter Group B: no antiseptic catheter	CA-UTI incidence of group A vs group B: OR 0.90 (95% CI 0.73-1.10	There were no differences in CA-UTI rates, although methodological issues hamper the generalizability of this finding. Antibacterial agents may prove to be significant in a well-conducted study. The present results provide good evidence to inform infection control guidelines in catheter management.	
Studies on preventative and/ or educational initiatives							
Loeb et al., 2008 [55]	RCT	To assess whether stop orders for indwelling urinary catheters reduces the duration of inappropriate urinary catheterization and the incidence of urinary tract infections.	patients admitted to hospital with indwelling urinary catheters inserted for ≤ 48 h.	Group A (n=347): Stop order group Group B (n=345): usual care group	Group A: 51/269 = (19%) Group B: 51 /252= (20%) RR 0.94, (95% CI, 0.66 to 1.33	Stop orders for urinary catheterization safely reduced the duration of inappropriate urinary catheterization in hospitalized patients but did not reduce urinary tract infections.	
Meddings et al., 2010 [56]	SR and MA on interventional studies (trials and pre-/post-)	To summarize the effect of urinary catheter reminder systems on the rate of CA-UTI, urinary catheter use, and the need for recatheterization.	catheter removal in hospitalized adults	14 studies included interventions (of a reminder or stop order) to remind treating doctors or nurses to remove unnecessary urinary catheters	The rate of CA-UTI (episodes per 1000 catheter-days) was reduced by 52% (P < .001) with the use of a reminder or stop order.	Urinary catheter reminders and stop orders appear to reduce the rate of CA-UTI and should be strongly considered to enhance the safety of hospitalized patients.	
Chen et al., 2013 [57]	RCT	To determine whether a use of the criteria-based reminder system would reduce the use of urinary catheters and the incidence of catheter-associated urinary tract infections.	278 patients from 2 respiratory intensive care units with indwelling urinary catheters. Patients who had urinary catheters in place for more than 2 days from April through November 2008 were randomly assigned.	Group A (147 patients): (Intervention group) use criteria-based reminder criteria to remove the catheter. Group B (131 patients): (Control group) no removal criteria	Number of CA-UTI cases: Group A: 20 (13.6%) Group B: 34 (25.9%) RR 0.52 ((0.32-0.86), p<0.01)	The use of a criteria-based reminder to remove indwelling urinary catheters can diminish the use of urinary catheterization and reduce the likelihood of catheter-associated urinary infections.	
Lee et al., 2015 [58]	RCT	to evaluate the effects of a nurse–family partnership model on the self-efficacy of family caregivers (FCs) and the incidence of CA-UTI	patients with an indwelling urinary catheter	Group A (30 patients): nurse attended a 4 h training course Group B (31 patients): routine nursing care	Number of CA-UTI incidences: Group A: 6 (20%) Group B: 12 (38.7%)	Our study considered caregivers as partners in caring for patients with indwelling catheters, and we examined an intervention to enhance the self-efficacy of FCs in urinary catheter-associated care to reduce the occurrence of CA-UTIs. The results showed that the effects of the intervention did not differ statistically. The self-efficacy of caregivers and the occurrence of CA-UTIs in patients in the two groups were statistically equivalent.	
Mody et al., 2015 [59]	RCT	To test whether a multimodal targeted infection program (TIP) reduces the prevalence of multidrug-resistant organisms (MDROs) and incident device-related infections.	418 Participants were high-risk NH residents with urinary catheters, feeding tubes, or both.	Group A: intervention: Multimodal, including (1) preemptive barrier preCA-UTIons, (2) active surveillance for MDROs and infections, with data feedback, (3) NH staff education on key infection prevention practices and hand hygiene promotion. B: control group	Urinary catheter number of cases: A: 120 (59.1%) B: 32,6 % First new clinically defined CA-UTIs in 166 residents: HR 0.54 (0.30-0.97) All (including recurrent) clinically defined: HR 0.69 (95% CI, 0.49-0.99)	Our multimodal targeted infection program intervention reduced the overall multidrug-resistant organisms (MDRO) prevalence density, new methicillin-resistant S aureus acquisitions, and clinically defined catheter-associated urinary tract infection rates in high-risk NH residents with indwelling devices.	
Wilde et al., 2015 [60]	RCT	to determine the effectiveness of a self-management intervention in the prevention of adverse outcomes	202 adult long-term urinary catheter participants	Group A: learning catheter-related self-monitoring and self-management skills during home visits by a study nurse Group B: usual care by home care nurses, clinics, or private providers)	The baseline CA-UTI rate of 6.93/1000 catheter days decreased to 4.89 (a 29% relative reduction) and in the control group from 5.5/1000 catheter days to 4.12 (a 25% relative reduction;	A simple-to-use catheter problems calendar and the bi-monthly interviews might have functioned as a modest self-monitoring intervention for persons in both groups. A simplified intervention using a self-monitoring calendar is suggested-with optimal and consistent fluid intake likely to add value.	
Durant et al., 2017 [61]	SR on case-control studies	to systematically evaluate the effectiveness of Nurse-driven protocols (NDPs) in preventing CA-UTIs.	29 studies primarily focused on intensive care units and several others in academic hospitals.	Group A: NDPs Group B: normal practice	all reported reductions in clinical predictors of CA-UTI, particularly indwelling-urinary catheter utilization and CA-UTI rates.	NDPs appear to have a positive impact on the clinical predictors and prevalence of CA-UTI. However, this review identified the need for improving the study design of quality improvement projects conducted within the patient care setting.	
Gould et al., 2017 [62]	SR on RCT & non-RCT	To establish whether implementing clinical guidelines can reduce infection rates in long-term care or improve the quality of urinary catheter care in nursing home settings.	nursing homes residents or in care facilities	Implemented clinical guidelines.	Three studies evaluated the impact of implementing a complete clinical guideline, all three reported reductions of CA-UTI. Five additional studies evaluated the impact of implementing individual elements of a clinical guideline, Hazard ratio for CA-UTI was significantly reduced in the intervention group compared to the control.	Prevention of catheter-associated urinary tract infection in nursing homes has received little clinical or research attention. Studies concerned with whole guideline implementation emerged as methodologically poor using recognized criteria for critically appraising epidemiologic studies concerned with infection prevention. Research evaluating the impact of single elements of clinical guidelines is more robust, and their findings could be implemented to prevent urinary infections in nursing homes.	
Meddings et al., 2017 [63]	SR on comparison studies	Identify strategies to reduce UTIs in nursing home residents.	20 records describing 19 interventions were included: 8 randomized controlled trials, 10 pre-post nonrandomized interventions, and 1 nonrandomized intervention with concurrent controls. nursing home residents participated	Interventions involving urinary catheter use such as improving appropriate use, aseptic placement, maintenance care, and prompting the removal of unnecessary catheters.	The 19 studies reported 12 UTI outcomes, 9 CA-UTI outcomes, 4 bacteriuria outcomes, and 5 catheter use outcomes. Five studies showed CA-UTI reduction (1 significantly); 9 studies showed UTI reduction (none significantly); 2 studies showed bacteriuria reduction (none significantly). Four studies showed reduced catheter use (1 significantly).	Several practices, often implemented in bundles, appear to reduce UTI or CA-UTI in nursing home residents such as improving hand hygiene, reducing and improving catheter use, managing incontinence without catheters, and enhanced barrier preCA-UTIons barrier enhanced preCA-UTIons	
Potugari et al., 2020 [64]	SR	To compare the catheter-associated urinary tract infections (CA-UTI) standardized infection rate (SIR) before and after implementation of a multimodal intervention approach in a rural tertiary hospital.	Patients admitted for in-patient care.	Before-after analysis of a multimodal intervention to evaluate primary outcomes of the incidence of inpatient CA-UTI, the SIR for CA-UTI, and the number of urinary catheter days.	CA-UTI event rates decreased, and SIR for CA-UTI was reduced by 60.2% (from 1.524 to 0.607) with a p value<0.05.	Incidence of CA-UTIs was significantly reduced with a team effort involving infection control, physician and nursing education, modification of progress notes and templates and daily provider reminders for the clinical necessity of catheters and appropriate usage of a urinary catheters with the corresponding reduction in urinary catheters days.	
Miscellaneous studies concerning UTI:							
Phipps et al., 2006 [65]	SR Cochrane of RCT and quasi-RCT	To establish the optimal way to manage urinary catheters following urogenital surgery in adults	Adults undergoing urogenital surgery.	1.All urinary catheterization, by urethral, suprapubic or both routes; • 2.use of 2-way or 3-way catheters of all sizes 3. use of PVC, silicone or latex catheters 4. use of bladder irrigation and/or wash-out. 5. use of policies regarding postoperative timing of catheter removal 6. use of policies regarding bladder filling prior to catheter removal manipulation 7. use of policies regarding the time of day of catheter removal 8. use of antibiotic policies regarding catheter 9. use of clamping or catheter prior to removal; 10. use of post-void residual volume measurement prior to suprapubic catheter removal 11. use of policies for assessment following catheter removal.	Using or not using urinary catheter: The data from five trials were heterogeneous but tended to indicate a higher risk of re-catheterization if a catheter was not used postoperatively. The data gave only an imprecise estimate of any difference in urinary tract infection. Urethral or suprapubic catheterization: In six trials, recatheterised was more if a urethral catheter rather than a suprapubic one was used following surgery (RR 3.66, 95% CI 1.41 to 9.49). Long versus short duration: seven trials suggested fewer urinary tract infections when a catheter was removed early. Clamp versus immediate catheter removal: one small trial concluded that more incidence of urinary tract infections resulted from the clamp and release group (RR 4.00, 95% 1.55 to 10.29).	Despite reviewing 39 eligible trials, few firm conclusions could be reached because of the multiple comparisons considered, the small size of individual trials, and their low quality. Whether or not to use a particular policy is usually a trade-off between the risks of morbidity (especially infection) and risks of recatheterisation.	
Kringel et al., 2010 [66]	RCT	Evaluating different protocols of postoperative drainage	Patients of anterior colporrhaphy plus an optional further procedure (i.e., hysterectomy)	Group A (n= 100): transurethral catheter for 24h Group B (n= 100): transurethral catheter for 96h Group C (n= 32 ): suprapubic catheter for 96 h	Number of UTI in each group: A: 2 B: 6 C: 0 (p=0.155)	The optimal bladder catheter after anterior colporrhaphy was, in our trial, the IUC for 24 h.	
Abdel-Aleem et al., 2014 [67]	RCT	To assess the effectiveness and safety of indwelling bladder catheterisation for intraoperative and postoperative care in women undergoing CS.	women undergoing CS (planned or emergency)	A: Indwelling bladder catheter during and after CS versus no catheter B: Indwelling bladder catheter during and after CS versus bladder drainage	Indwelling bladder catheterisation was associated with a reduced incidence of bladder distension (non‐prespecified outcome) at the end of the operation (risk ratio (RR) 0.02, 95% confidence interval (CI) 0.00 to 0.35; one study, 420 women) There was no difference between groups (RR 1.27, 95% CI 0.58 to 2.77; 225 women. There was also no difference in the incidence of UTI (as defined by trialists) between the indwelling bladder catheterization and no catheterization groups (two studies, 570 women).	This review includes limited evidence from five RCTs of moderate quality. The review's primary outcomes (bladder injury during operation and UTI), were either not reported or reported in a way not suitable for our analysis. The evidence in this review is based on some secondary outcomes, with heterogeneity present in some of the analyses. There is insufficient evidence to assess the routine use of indwelling bladder catheters in women undergoing CS. There is a need for more rigorous RCTs, with adequate sample sizes, standardised criteria for the diagnosis of UTI and other common outcomes.	

Route of administration and catheterization selection type 

Comparing Intermittent Catheterization & Short-term Indwelling Catheters?

Two RCTs [9,10] and two SR [11,12] were identified in which intermittent (self-) catheterization was compared with the use of temporary indwelling catheters. Including the 182 patients who underwent hip fracture or hip replacement surgery that Hälleberg Nyman et al. included; The absolute risk difference of CA-UTI in the intermittent catheterization group was a low 2.4% with a Confidence interval of 6.9 to 11.6%, a statistically insignificant difference (8 out of 85 patients (9.4%) with CA-UTI in the intermittently catheterized group, compared to 10 out of 85 patients (11.8) with indwelling catheters [10]. When comparing transurethral indwelling with intermittent catheters, Hakvoort et al. reported a (p = 0.03) lower CA-UTI rate with intermittent catheterization (12% rate) compared to an indwelling catheter (33%) left in place for seventy-two hours [9]. 

Nine RCTs with 1771 patients were included in a meta-analysis by Zhang et al. There was no significant difference in the rate of UTIs between indwelling catheterization and intermittent catheterization groups (RR: 1.23; CI 95% [0.85; 1.76], P>0.05). At the same time, Hunter et al. concluded that the evidence was equivocal for symptomatic urinary tract infection [11]. 

Comparing Temporary Transurethral to Suprapubic Catheterization

Two RCTs compared the use of transurethral and suprapubic catheterization in patients who required urological interventions; suprapubic catheterization was comparable to transurethral catheterization with little to no difference in the rate of infection [13,14]. Two SRs [12,15] and one Cochrane review [16] were also identified. When comparing suprapubic to urethral, all revealed that there is no statistically significant difference in the rates of CA-UTI. In a systematic review and meta-analysis (included twelve RCTs) by Healy et al., suprapubic catheterization was associated with a significant reduction in postoperative UTIs (20%; OR: 0.31; 95% CI, 0.185-0.512; p < 0.01) compared to 31% for urethral catheterization in the selected gynecologic patients [15]. Patients are three times more likely to develop a UTI with a transurethral catheter than a suprapubic catheter. Although Healy pointed out the increased noninfectious complication rate that suprapubic catheter was associated with (29% compared to 11%; OR: 4.14; 95% CI, 1.33-12.9; p = 0.01), those were tube malfunction related with no visceral injuries reported among the 1,300 participants [15]. 

Hunter et al. identified studies comparing suprapubic catheterization to various other methods of chronic bladder emptying, such as intermittent or indwelling urethral catheterization in the adult population [12]. Their review focused on suprapubic catheters, with the available evidence of 14 studies (one prospective non-randomized study and eight retrospective reviews with a comparator, a case-series, and qualitative assessments of quality of life) reported no significant difference between symptomatic CA-UTI outcomes between suprapubic and urethral catheters. However, the evidence is limited by the varied UTI criteria defined in their outcomes. The study revealed that suprapubic catheterization was associated with a lower incidence of urethral complications. However, the incidence of upper and lower urinary tract complications between urethral and suprapubic catheters was similar [12]. Similarly, a Cochrane review by Kidd et al. found an insufficient difference in symptomatic UTI risk between the suprapubic versus indwelling urethral catheters, but the suprapubic catheter group were catheterized for a longer duration than the urethral group (RR: 1.01; [0.61; 1.69]) [16]. 

However, a Cochrane systematic review comparing short-term indwelling urethral catheters to suprapubic catheters found that indwelling catheterizations lead to more incidents of bacteriuria (RR 2.6, 95% CI 2.12, 3.18) and patient discomfort (RR 2.98; 95% CI 2.31, 3.85)[52]. 

*Fixation with a Catheter Securing Device (StatLock ®) or Valve* 

Only one review devoted to reviewing the evidence on the effect of catheter valves compared to free drainage into a bag for patients with indwelling urinary catheter [18]. While two RCT were evaluated by Van den Eijkel et al., results relating to CA-UTI were only reported in a single RCT; 60% of the intervention group with the valve developed CA-UTI, compared to 68% in the control group. The absolute differential in the infection rates of the two groups was statistically insignificant, with a p = 0.286 [17].

An RCT conducted by Darouiche et al. investigated the effect of the StatLock ® system, a fixation device for indwelling catheters was used in adult patients with neurogenic bladder due to spinal cord injury. Out of the 118 patients, among the 60 patients who received StatLock, the rate of CA-UTI was 45% lower, but a statistically insignificant association with increased UTI rates was established (RR: 0.55; [0.25; 1.22]) [18]. 

*Bladder Clamping and Free Urinary Drainage* 

One RCT [19] and three SRs [20,21,65] evaluated the necessity of bladder clamping before removing a urinary catheter. The RCT by Gong et al. did not report any statistically significant difference between the two groups (CA-UTI in the clamping group was 22.9% vs 20.3% for the controlled group). Wang et al. revealed no statistically significant difference between clamping and free drainage. There was no significant difference between clamping and unclamping groups found across four studies included in their study (OR 0.76, 95% CI (0.33, 1.73)). Gong et al. and Wang et al. concluded no significant difference between the clamping and unclamping groups in the outcomes of UTI and patients bladder function [19,21]. 

Fernandez et al. [20] delineated and compared three timepoints of catheter management as follows: (Group A) within 24 hours removal after free drainage, (Group B) within 72 hours removal, (Group C) within 24 hours removal in addition to bladder re-education. No significant differences were reported in the rates of CA-UTI in the 24 hours group (RR: 1.12 [0.24; 5.18]) or in the 72 hours group (RR: 0.55; [0.15; 2.01]). There was no statistically significant difference in the UTI rates for clamping compared to free drainage for 24 or 72 hours before catheterization removal. In contrast, a Cochrane review that included one study favoured free immediate catheter removal with RR 4.00 (1.55, 10.29) [20]. 

Duration of catheterization and minimizing dwell time 

The time a catheter is in place for a particular time associated with operative gynaecological interventions was studied in seven RCTs [22-28]. The catheters in the trials were either immediately removed postoperative or within 24 hours in the following RCTs [22-25]. While Bray et al. set the catheter removal 48 to 72 hours after as the prerequisite criteria to their intervention, Weemhoff et al. compared catheterization duration of two vs five days and its association with temporary catheter replacements, temporary catheter replacements and fewer urinary tract infections, and shorter hospital stays [27,28]. The seven authors of the selected RCTs concluded that the policy of short term catheterization compared to immediate or long term catheterization was associated with lower symptomatic urinary tract infection rates and less rate of re-catheterization. All agreed that short term catheterization is also associated with earlier postoperative ambulation. However, all the studies but Chai et al. did not report lower rates of CA-UTI [24]. Moreover, contrary to the data above by the other authors, Liang et al. concluded that short term indwelling catheterization increased the incidence of UTI [30]. 

Phipps et al. conducted a Cochrane systematic review dealing with transurethral and suprapubic catheterization focusing on the following durations of catheterization: one vs. two days (one study: RR: 0.52; [0.05; 5.40]), one vs. three days (three un-pooled studies [RR: 0.11; (0.03; 0.43)]), one day vs. five days (two un-pooled studies, RRs 0.11 [0.03, 0.43]; 0.70 [ 0.29, 1.67]), and one vs. 14 days (one study, RR 0.21 [0.03, 1.65]) [65]. Out of the 11 trials, seven trials with data suggested fewer urinary tract infections when a catheter was removed earlier. Although the studies did not indicate any statistical significance in the confidence interval of CA-UTI incidences, the point estimators conclusively indicated that the shorter duration resulted in the overall better outcome [65].

Fernandez et al. was the one study that purely focused on the duration and set different catheterization durations before the removal of short-term indwelling urethral catheters [30]. Four trials out of the eight showed no significant differences in CA-UTI rates in patient outcome after TURP (RR 0.55, 95% CI 0.30 to 1.03). A statistically significant difference in CA-UTI rate was reported in one RCT that compared catheterization for 1 vs 5 days after rectal resection. The five-day rate was almost as twice as higher as the one-day indwelling catheterization [30]. Similarly, Phipps et al. suggested fewer urinary tract infections occur when a catheter is removed earlier (for example, 1 vs 3 days, RR 0.50, 95% CI 0.29 to 0.87) [65]. 

It is worth noting that both the Lam et al. and Lusardi et al. reviews evaluated the expected duration of internal bladder catheters for up to 14 days. Whether they including a large number of interventions to be evaluated affected the calculated incidence rate of urinary tract infection in their studies or not remains to be unknown [38,39]. 

Assessing indication/necessity for catheterization

Li et al. suggested that the routine use of indwelling urinary catheters for caesarean delivery is not necessary and is associated with fewer UTIs and no increase in postoperative adverse urinary events (relative risk [RR] of urinary tract infection compared with the use of indwelling urinary catheters, the non-catheterized patients had a significantly lower incidence of UTIs [RR 0.08; with 95% confidence interval 0.01, 0.64 (study design: RCT); the RR in the single NCRT was 0.10 with 95% CI 0.02, 0.57] [37]. 

Regarding the incidence of UTIs in another cesarean-related study, Nasr et al. found no statistically significant difference (P<0.001) between the catheterized and non-catheterized patients regarding UTI symptoms. The incidence of UTIs was 5.7% in the catheterized group for the 24 h postoperative and 2.9% for the one week postoperative, vs 0.5% (P<0.001) 24 h postoperative and 0% (P<0.001) one week after the operation in the non-catheterized [36]. 

Maintenance and care of catheterized patients 

Cleansing or Disinfection of the External Urethral Orifice 

A network meta-analysis by Ercole et al. summarized data from thirty-three studies with seven different methods of urethral cleaning versus disinfection of the external urethral orifice was included (normal saline vs tap water vs soapy water vs antibacterial vs iodine vs chlorhexidine) [31]. No evidence of heterogeneity (P>0.05) was observed among the studies. The results showed no statistical difference in the incidence of CA-UTIs (P>0.05 for all) when analyzing the different urethral cleaning methods versus disinfection [31]. 

Cao et al., through twenty-eight RCTs and nine SRs, that they included in their review, presents that the rate of urinary tract infection is not predicated on whether the perineum is cleaned with or without sterile water, or with the use of the povidone-iodine solution or chlorhexidine, or even with the use of clean or sterile technique, no difference in the incidence of CA-UTIs when comparing the different urethral cleaning methods versus disinfection (P > 0.05 for all), this was postulated based upon thirty-three trials including 6390 patients with seven different urethral cleanings versus disinfection methods [32]. Similarly, studies that, prior to intermittent or indwelling catheterization, used anti-septic or non-medicated agents to clean peri-urethral or meatal areas showed no statistical significance in reducing its association with the incidence rate of UTI [33]. 

It was suggested that cleaning the peri-urethral area before catheter insertion can be undertaken, non-sterile water would be an equally weighted option, and the economical alternative as its effectiveness compared to the sterile water. Anti-septic solutions (chlorhexidine and PVP-I) were as equal. More studies about UTI development and saved expenditure on costs were not looked at and needed to be confirmed [31-33]. 

Even though a consistent level of hygiene was scrutinized with short-term catheter use in their RCT, Fasugba et al. failed to result in a substantial reduction in CA-UTI rates for long-term catheterization [54].

Irrigations and Washouts 

The practice of irrigating long-term indwelling urinary catheters has also been assessed by two systematic reviews [34,35], including reports of various solutions and regimens. One of the RCTs in a dedicated RCT conducted by Shepherd et al. [35]. Four trials studied the following: (any washout vs no washout, saline washout versus no washout, citric acid washout versus no washout). The authors were uncertain if comparing washout and no washout had any significant effect on the rate of symptomatic UTI or duration of catheterization in situ. The evidence was not adequate to conclude if washouts were beneficial or harmful due to the poor methodological quality and reporting [35].

Both Systematic reviews have five studies that were labelled to be of poor quality and concluded inconclusive effective at either reducing symptomatic CA-UTIs or duration of first catheter change [34,35]. 

Prophylactic measures 

*Antiseptic-coated Compared to Standard Non-septic Catheters* 

Lam, Pickard, Bonfill, and Jahn, most notably, from the studies we included, compared the effect of anti-septic catheter surfaces vs the non-septic catheters, respectively [38,40-42]. Two Cochrane reviews conducted by Lam et al. and Jahn et al. have not revealed any advantageous benefits of silver-coated catheters over the standard one (4241 patients; RR: 0.99; [0.85; 1.16]) and (20 patients; RR: 10; [0.83; 1.2]) respectively [38,42]. Jahn et al. concluded evidence should not be treated as a reliable basis for practical implications due to the small sample of the trials and that very few trials have compared several types of catheters for long-term bladder drainage [42]. 

While Lam et al. [38] concluded that the antiseptic-coated catheters resulted in no statistically significant reduction in symptomatic CA-UTI and was considerably expensive, Pickard'sPickard's 2012 RCT further stated that silver alloy-impregnated catheters might be less cost-effective than the antibiotic (Nitrofurazone) coated with OR 0.96 (0.78 to 1.19); p=0.69 compared to OR 0.81 (0.65 to 1.01) p=0.031, respectively [40]. 

There is no unequivocal evidence supporting the use of either anti-septic, or antimicrobial coated catheters is more beneficial than using standard catheters in reducing UTI in patients who require long term catheterization, no sufficient data to decide which type is the go-to for CA-UTI prevention [41,42]. 

*Antiseptic-coated Compared to Antibiotic-impregnated* 

One large trial included in a Cochrane review SR from 2014 compared silver alloy-coated (antiseptic-coated) catheters versus antimicrobial-coated (nitrofurazone) catheters; they suggested an advantage of antiseptic-impregnated catheters over nitrofural-impregnated catheters (one study; 4250 patients; RR: 0.84; [071; 1.00]) [38]. The results showed that people were less likely to have asymptomatic CA-UTI with nitrofurazone-impregnated (228 in 2153 patients, 10.6%) than silver alloy-coated (263 in 2097 patients 12.5%). However, the magnitude of reduction was not statistically significant and hence may not be clinically important (RR 0.84, 95% CI 0.71 to 1.00) [38]. 

Beattie et al. emphasized that the heterogeneity was too significant for them to calculate an estimate for all studies combined but stated that there was nothing to suggest that one approach was better than the other [43]. The low number of participants, wide confidence intervals and risk of systematic errors and biases in one of the studies means that the methodological quality should be considered to be a low one, and cannot conclude if whether silver-alloy urinary catheters reduce CA-UTI compared with standard silicon or latex urinary catheters [43]. 

Antibiotic-related Prophylaxis 

Either comparing prophylactic antibiotic administration with no antibiotic prophylaxis or using antibiotic-impregnated catheters were discussed in eleven studies; five are RCTs [40,44-48], four are SRs [38,39,47,52], and four Cochrane Database SRs [38,39,43,52]. In one trial, conducted by Niël-Weise et al., in comparing antibiotic prophylaxis with antibiotics administration when clinically indicated in the female surgical patients who had a urethral catheter for more than 24 hours, symptomatic UTI was less frequent in the prophylaxis group (RR 0.20, 95% CI 0.06 to 0.66) [52]. Likewise, Berrondo et al., in their prospective, randomized, controlled trial, using antibiotic prophylaxis with oral ciprofloxacin before urinary catheter removal after radical prostatectomy did not decrease UTI rate [49]. 

Moreover, in adults requiring short-term urinary urethral and supra-pubic catheterization up to and including 14 days, the patients who received the following systemic antibiotic prophylaxis (cefotaxime, trimethoprim/sulfamethoxazole, ciprofloxacin, or Nitrofurantoin) antibiotic prophylaxis was associated with an absolute reduction in risk of urinary tract infection of 5.8% with a risk ratio of 0.45 and a 95% CI between 0.28 to 0.72) [47]. Marschall et al. reported a number needed to treat of 17 (95% confidence interval, 12 to 30) to prevent a single CA-UTI [47]. However, Van Hees et al. concluded that their results do not support antibiotic prophylaxis for urinary catheter removal in non-genitourinary surgical patients. Their study included patients who underwent surgery and received a single prophylactic antibiotic dose 2hours before catheter removal (ciprofloxacin 500 mg [n = 43], co-trimoxazole 960 mg [n = 46], placebo [n = 51]) [48]. 

Lam et al. reported a significant difference in the use of nitrofural-impregnated catheters compared to standard catheters (one trial that included 4297 patients concluded a RR of 0.84 with a 95% confidence interval of [0.71; 0.99]) [38]. While Lusardi et al. for the systemic intravenous administration of trimethoprim/sulfamethoxazole (single trial; 90 patients; RR: 0.20; [0.06; 0.66]) [39]. 

*Phytotherapeutic Cranberry Extracts* 

Both Foxman et al. and Gunnarsson et al. reviewed whether the prophylactic use of cranberry extract tablets during the postoperative period will reduce or even prevent the occurrence of CA-UTI [50,51]. In the study by Gunnarson et al., 227 female patients, aged 60 years and older, with hip fractures were randomized to either receive 550 mg of cranberry powder three times daily or placebo capsules daily until five days postoperatively [51]. There was no difference between the groups of patients with postoperative positive urine cultures at either day 5 or 14 days postoperatively (p = 0.975): 13 of 33 (39%) in the placebo group and 13 of 47 (28%) in the cranberry group (P=0.270) had a positive urine culture. However, this difference was not statistically significant (P=0.270) [51]. 

Foxman et al. concluded that patients undergoing elective gynecologic surgery involving urinary catheterization, the use of cranberry tablets during the postoperative period reduced the rate of UTI by at least a half; 15 of 80 patients (19%) for the intervention group in comparison to 30 of 80 patients (38%) for the placebo group with positive urine culture; (OR=0.38; 95% CI: 0.19, 0.79; p=0.008) [50]. 

Educational Protocols & Preventative Implementations 

Educational and raising patient awareness approaches were discussed in eleven studies, five SRs [56,61-64] and five RCTs [55,57-60]. Meddings et al., who reviewed catheter discontinuation strategies for hospitalized patients and pooled their results of 7 seven trials, reported that the "stop order" intervention to prompt removal of unnecessary catheters reduced the duration of catheters in place by 1.06 days, and the use of either "reminders or stop orders" decreased the CA-UTI rate by 53%, (RR: 0.48; [0.28; 0.68]; p = 0.001) [56]. Another review of nineteen studies by Meddings et al. reported that CA-UTI decreased with compliance with hand hygiene protocols during urethral catheter administrations and any follow-up catheter cares [63]. While Wilde et al. determined in their study in the experimental group (learning catheter-related self-monitoring and self-management skills during home visits), the baseline CA-UTI rate of 6.93/1000 catheter days decreased to 4.89 with a 29% relative reduction while in the control group from 5.5/1000 catheter days to 4.12 with a 25% relative reduction [60]. 

Another systemic review by Mody et al. confirmed that the efficacy of implementing preventive protocols in nursing homes did reduce the catheter-associated UTI rates decreased from 6.78 to 2.63 infections per 1000 catheter-days [59]. With use of the random-effects negative binomial regression models, the rates decreased from 6.42 to 3.33 (incidence rate ratio [IRR], 0.46; 95% CI, 0.36-0.58; P < .001) [59]. This was the only intervention that demonstrated a statistically significant reduction in CA-UTI in chronically catheterized patients due to the implemented comprehensive program that limited antimicrobial use, improved hand hygiene, and promote standardized CA-UTI definitions and active drug-resistant organisms surveillance protocol [59]. 

Miscellaneous & Coupled Interventions 

One Cochrane review, following urogenital surgery in adults, examined seven trials that compared the postoperative duration of catheter use; these trials suggested that shorter-term catheterization was associated with fewer UTI incidences and more patients required re-catheterization following a urethral compared to a suprapubic catheter [65]. While Ercole et al. showed that the use of an intermittent catheter with clean technique results in low rates of complications or infections compared to the use of an indwelling catheter, in the same review, postoperative catheter removal up to 24 hours and the use of an antimicrobial-impregnated or hydrophilic-coated catheter resulted in lower urinary tract infection [31]. 

One RCT conducted by Kringel et al. reported that transurethral catheters left in for 24 hours cause lower infection compared to suprapubic catheters left in for a longer period of around 96 hours (p = 0.034) [66].

Discussion 

We included fifty-nine combined studies, including thirty-six RCTs and twenty-three SRs, meant to shed light on more than fifty measures. Ten of these measures were studied. The bulk of the studies was published in the 2010s period. Since we identified numerous studies with different evidence and measures regarding the prevention and management of CA-UTI in different patient groups and settings, the data regarding catheterization, duration, and prophylactic measure are clinically heterogeneous states of the evidence not conclusive. Although several review authors have identified a couple of studies for some interventions, they could not perform meta-analyses due to the highly heterogeneous finding between the included studies. Three contributing factors for the resulted heterogeneity might be as follows: 

Study Groups 

The patient groups that were compared varied from one study to the other. Patients with different diagnoses, anatomies and health/immunological status may have different preconditions and predispositions to infections, which in turn makes it harder to combine and group studies to obtain enough participants with similar physical and immunological conditions in comparison to be able to report an efficient data for the prevention of catheter-associated urinary tract infections. 

*Interventions* 

Different interventions lead to large variations in what was compared. Studies that were conducted before the first half of the first decade in the 2000s were excluded due to the fear that the applicability and practicality of their findings-in particular, relating to the guidelines related to administration of prophylactic antibiotics or types of education programs would not resonate nor translate to the current clinical practices; as the perception about prescribing practices and awareness around resistance may well have over the time. Therefore, to avoid the need to examine the preparations or methods used at the time in the study today or whether there have been other changes over the years, 2005 was agreed on. 

Measurement of Outcome Measures 

The terms Urinary tract infection, bacteriuria and catheter-associated urinary tract infection were loosely used and varied between the studies. Some studies have not even defined the criteria for their used outcome. Some studies used the terms CA-UTI and catheter-associated asymptomatic bacteriuria or catheter-associated bacteriuria interchangeably. Additionally, terms like Bacteriuria and UTI were differently defined. If we look at three publication as an example, definitions of urinary tract infection in those studies, for instance, were: > = 105 CFU/ml with> 10 leukocytes per mm3 of urine [38], > = 105 CFU/ml with one of the following symptoms: fever, pyuria, hematuria, chills, and/or dysreflexia [39], > = 105 CFU/ml [42].

All Catheter types are susceptible to biofilm formation and catheter encrustation; hence administering prophylactic antibiotics may delay the pathogenesis of CA-UTIs rather than preventing their occurrence. The use of low-dose, prophylactic antibiotics might aid in creating "persister" cells that are genetically capable of invading the uroepithelium and result in infection and integration with urinary bladder microbiota. Therefore, contributing more to sepsis and infection [68]. 

The duration of a catheter is generally based on individual cases rather than evidence-based knowledge and therefore varies among clinical practice. In-dwelling catheters have been associated with positive urine cultures, which can subsequently lead to urinary tract infection can which, as a result, increases the duration of hospital stay, costs and risk of morbidity. The risk of developing a CA-UTI is related to catheter dwell time [69,70]. For catheterized patients, the rate of development of catheter-associated bacteriuria is between 3% to 7% per day [71,72]. The likelihood of bacteriuria approaches 100% if a patient has an indwelling urinary catheter for ≥30 days [73,74], which is part of the rationale for why a urine culture alone is not sufficient to diagnose a CA-UTI. While bacteriuria is a risk factor for UTI, the frequency of progression from bacteriuria to CA-UTI is low and treating ASB does not decrease the risk of future CA-UTI. Other risk factors for the development of CA-UTI include urinary tract instrumentation, diabetes mellitus, and malnutrition [75,76]. The two principal factors that lead to CA-UTIs are unnecessary urinary catheter placement and unacceptable delay in removing a catheter when it is no longer needed [77]. Unfortunately, 38% of attending physicians are unaware that their patients have a urinary catheter in place, which might be due to the ambiguity of catheter placement indication in approximately 30% of cases [78]. 

An analysis by Hutton et al. [79] showed that implementing their multimodal intervention program led to 8.7 fewer CA-UTIs and 2.9 fewer resident hospitalizations per nursing home per year. 120-bed NH would have program costs of $20,279/year. The cost of disease treatment would be reduced by $54,316 per year, resulting in a net cost savings of $34,037. A cost savings of $15,136 in CA-UTI care and $39,180 in-hospital care for CA-UTIs and CA-UTI-associated septicemia, for a total net savings of $34,037 for the healthcare system. As well as 0.2 more QALYs (quality-adjusted life-years) than their control group. 

Duszyńska et al. estimated the cost of HAIs in a Polish ICU to range from EUR 10,035 to 22,411 [80]. While in the USA, an estimate of 449,334 healthcare-associated catheter-associated urinary tract infections (CA-UTIs) per year, associated with an additional cost of US$749-10077-9 per admission in 2007 (or an estimated US$3744 when complicated by blood septicemia) [77,81]. 

An Australian study revealed that staffing costs for infection prevention nurses exceed AUD 100million per year and that 36% of their time is spent on patient monitoring. Another study confirmed that those undertaking active surveillance on patients had never been trained, and skills like reporting data to hospital executives are either not appropriately done [82,83]. This means that much of the CA-UTI data being collected might not be a true reflection of the magnitude of catheter complications and makes it harder to analyze the infection rate in an efficient, productive manner. Saint et al. surveyed 719 acute-care American hospitals for their CA-UTI prevention protocols in 2005; more than 70% of the surveyed hospitals documented their rates of CA-UTI, 44% documented which patient had a urinary catheter inserted, and 26% documented the duration of catheterization. No widely accepted protocol to prevent CA-UTI was reported. 30% of the surveyed hospitals reported the regular use of antimicrobial catheters, 14% reported condom catheters in men, and a mere 9% used catheter reminders or stop-orders [84]. 

## Conclusions

In terms of implications to clinical practice, the results of this review suggest that healthcare workers should think of 2 strategies to reduce rates of CA-UTI: limit catheter use and shorten the duration of catheterization. The literature also supports either daily scheduled reviews or stop orders to safely reduce the duration of inappropriate urinary catheterization in hospitalized patients. Based on the current state of evidence, there are insufficient data to determine whether transurethral or suprapubic routes are most appropriate for catheterization. The reduced morbidity rate of suprapubic catheterization is offset by higher rates of catheter-related complications and doesn't necessarily mean a shorter hospital stay. No good evidence exists to adequately conclude if washouts were beneficial or harmful due to poor methodological quality and the substantial risk of bias of the included studies. No significant difference was found between the clamping and unclamping groups. Given the scant state of evidence, procedures relating to clamping of indwelling urinary catheters should not be favoured over free drainage. No significant differences have been demonstrated among the various methods of cleansing or disinfecting the external urethral orifice. Evidence from studies that, before intermittent or indwelling catheterization, used either anti-septic or non-medicated agents to clean peri-urethral or meatal area showed no statistical significance in reducing its association with the incidence rate of UTI. Evidence of antiseptically coated catheters, compared to standard uncoated catheters, is equivocal. Antibiotic-impregnated catheters seem to be more documented in the literature and reduce the rate of catheter-associated symptomatic urinary tract infection. The current evidence on phytotherapy using cranberry extracts to prevent UTIs remains debatable, in part due to the trials were small and methodological weaknesses were shown. Therefore, the evidence was not a reliable basis for any clinical conclusions. So, there is no well-justification in recommending it highly. 
